# Individuals’ Decision to Disclose a Diagnosis of Dementia and the Development of an Online Empowerment Intervention

**DOI:** 10.1093/geroni/igab046.2254

**Published:** 2021-12-17

**Authors:** Gianna Kohl, Mauricio Molinari Ulate, Jem Bhatt, Jennifer Lynch, Katrina Scior, Georgina Charlesworth

**Affiliations:** 1 University College London, University College London, England, United Kingdom; 2 University of Salamanca, Salamanca, Castilla y Leon, Spain; 3 University College London, London, England, United Kingdom; 4 University of Hertfordshire, Hatfield, England, United Kingdom

## Abstract

Learning to live with a diagnosis of dementia is a complex process. Many people affected by dementia choose not to disclose the diagnosis to others and avoid social activities due to fear of others’ adverse reactions. This in turn can limit their social participation and negatively affect their psychosocial health. A systematic review explored factors influencing the decision to disclose or conceal a dementia diagnosis to one’s social network, including individuals’ attitudes and experiences regarding this decision. The sixteen studies included reveal the complexity of this decision. Findings highlight the role of stigma and individuals’ wishes to remain ‘normal’, but also the need of explaining what has changed. Results were further discussed with people with dementia and informal caregivers as part of patient and public involvement. End users expressed their attitudes, needs, and wishes towards the design of an online empowerment intervention supporting disclosure decision-making in people affected by dementia.

